# Enhancement and analysis of Anthracene degradation by Tween 80 in LMS-HOBt

**DOI:** 10.1038/s41598-021-90609-5

**Published:** 2021-06-23

**Authors:** Zuoyi Yang, Xingchen Mao, Jiahao Cui, Yujie Wang, Yaping Zhang

**Affiliations:** Guangzhou Key Laboratory of Environmental Catalysis and Pollution Control, School of Environmental Science and Engineering, Guangdong University of Technology, Guangzhou, 510006 China

**Keywords:** Pollution remediation, Environmental sciences

## Abstract

This study examines the specific effect of Tween 80 on the conversion of anthracene (ANT) in laccase medium system regarding surfactant chemical changes and mechanism. The conversion rate and degradation products of ANT were investigated in different concentrations of Tween 80 solution. Between Tween 80 concentration 0–40 critical micelle concentrations (CMC), the kinetic parameter-k (h^−1^) and corresponding half-life T_1/2_ decreased with increasing concentration. When Tween 80 was above 20 CMC the laccase-medium system converted > 95% of ANT to anthraquinone within 12 h. During the entire enzymatic reaction, the laccase activity in the system increased with increasing Tween 80 concentration. Combined with GC/MS analysis of the product, it was speculated that hydrogens belonging to the ether-oxygen bond and carbon–carbon double bond α-CH of Tween 80, were removed by the laccase-media system, promoting its degradation. Additionally, enhanced activity caused by oxygen free radicals (ROS) such as RO• and ROO•, continuously oxidized Tween 80, which in turn produced free radicals while converting ANT. This study provides new theoretical support toward the application of surfactants in the elimination of polycyclic aromatic hydrocarbons.

## Introduction

Polycyclic aromatic hydrocarbons (PAHs) are volatile hydrocarbons produced by the incomplete combustion of coal, petroleum and organic macromolecules, and they are important environmental pollutants. They have caused continuous ecological and health issues, with a vast array of physical, chemical, biological as well as combinational technologies being developed to combat these environmental hazards^1^. Nowadays, bio-remediation technologies are employed to provide durable and low-cost solutions^2^. In this regard, compared with bacteria, fungi can be utilized in a wide range of substrates to produce extracellular enzymes, which are known to efficiently contribute to PAH degradation^3,4^. Furthermore, PAHs can be metabolized by a variety of fungal enzymes (such as lignin peroxidase and laccase), producing parent hydrocarbons with reduced toxicity^5^. In the case of laccase, it is regarded as a green biocatalyst and has recently received increased attention^6^.

The biocatalytic oxidation of laccase is limited owing to its low oxidation potential or that the molecular size of the substrate is incompatible with the enzyme’s active site^7^. Furthermore,2,2′-azino-bis(3-ethylbenzothiazoline-6-sulphonate) (ABTS) or 1- hydroxybenzotriazole (HOBt) is required due to the insolubility of PAHs in the medium. Generally, non-ionic surfactants (such as Tween 80 and Tween 20) can be also employed to increase PAH solubility^8–14^. However, the addition of surfactants to the laccase medium system (LMS) with PAHs increases its complexity^15^. Firstly, changes in enzyme activity and stability caused by interactions between surfactant and enzyme should be considered^16,17^. Secondly, PAH solubility enhanced by surfactant should not be ignored^18,19^.

However, the majority of studies focus on the physical effect of surfactants toward PAHs oxidation by LMS, while disregarding chemical changes of the surfactants during the enzymatic reaction. Reports have shown that the lipid peroxidation reaction of the unsaturated fatty acid structure in Tween 80 by reacting with LMS (HOBt medium) and involving hydrogen atom transfer (HAT) oxidation mechanism, can efficiently promote PAHs transformation^8,9^; additionally, the self-oxidation of Tween 80 or Tween 20 and production of reactive oxygen species (ROS) have also been confirmed in the field of medicine and food research^20–28^. However, the effect of surfactant oxidation on the degradation of target substances has rarely received attention in environmental research. Therefore, further studies are imperative in order to examine the effect of similar surfactant structures on PAH degradation by LMS.

In this present study, Tween 80 was chosen as the model non-ionic surfactant owing to its known high efficiency for the removal of hydrophobic organic compounds (HOCs) as well as enhancement of bioremediation and low environmental toxicity^29–32^. Accordingly, batch experiments were performed to investigate (i) the effect of Tween 80 on anthracene (ANT) degradation kinetic parameters (k) and half-lives (T_1/2_) by LMS with HOBt (LMS-HOBt), and (ii) degradation products and ROS of two non-ionic surfactants (Tween 80 and Tween 20) attained by LMS-HOBt allowing a proposed transformation mechanisms of ANT.

## Materials and methods

### Chemicals and materials

Laccase from *Trametes versicolor* (EC 1.10.3.2, CAS: 80498–15-3) was purchased from Sigma-Aldrich, USA; Anthracene (ANT) (analytical standard, CAS: 120-12-7), anthraquinone (ANQ) (analytical standard, CAS: 84–65-1), HOBt (≥ 97.0%, CAS: 80029-43-2), Tween 80 (cell culture grade, CAS: 9005–65-6), Tween 20 (viscous liquid, CAS: 9005-64-5) and HPLC-grade organic solvents including acetonitrile (CAS:75-05-8), *n*-hexane (CAS: 110-54-3) and methanol (CAS: 67-56-1) were obtained from Shanghai Aladdin, China. All solutions were prepared with the ultrapure water (Product model: Milli-Q Gradient, TOC < 1–5 ppb, 18.2 MΩ cm^−1^ resistivity).

### Transformation of ANT by LMS-HOBt in aqueous solution

Transformation of ANT (20 mg L^−1^) was investigated using 1.88 U mL^−1^ laccase and 2 mM HOBt. Reactions were performed in 0.2 M sodium acetate at pH 4.5, with a series of critical micelle concentrations (CMC) of Tween 80 (5, 10, 20, 30, 40 times CMC), which was reported as 13 mg L^−1^^33^, where 5 mL of the total reaction solution was placed in 20 mL Teflon-sealed vials under continuous shaking (150 r min^−1^) in darkness at 30 °C. At preselected time intervals (0–1 h at 20 min intervals, 1–4 h at 30 min intervals, 4–6 h at 1 h intervals, 6–12 h at 2 intervals. 12–24 h at 4 h intervals), the enzymatic reaction was terminated immediately with 5 mL acetonitrile, then the mixture was filtered through 0.22 μm Nylon 6 in order to determine ANT and ANQ (main transformation product of ANT by laccase or LMS)^10,12^ concentration by HPLC.

The residual ratio (R) of ANT in LMS was calculated using the following equation: R = *C*_t_ / *C*_0_, where *C*_t_ (mg L^−1^) is the residual ANT concentration measured at reaction time t, and *C*_0_ (mg L^−1^) is the initial ANT concentration at t = 0. To compare ANT transformation in the absence and presence of Tween 80, the transformation rate constants (*k*) of Tween 80 in aqueous solution were evaluated by the apparent pseudo first-order kinetics model, as shown in Eq. (1):

1 $$ {\text{ln }}\left( {C_{{\text{t}}} /C_{0} } \right) \, = \, - k\cdot{\text{t}} $$

where t is the reaction time, *C*_0_ is the initial concentration of ANT (t = 0), and *C*_t_ is the residual concentration of ANT at reaction time t.

The time course required the transformation of 50% of the initial ANT concentration which was calculated directly from *k* values using Eq. (2):

2 $$ T_{{{1}/{2}}} = {\text{ ln2 }}/k $$

Furthermore, Tween 20 and Triton X-100 were compared with Tween 80 in a series of concentrations (50, 100, 200, 400, and 600 mg L^−1^) under the same experimental conditions, in order to verify whether Tween 20 and Triton X-100 that contain ether oxygen bond, influence the transformation of ANT.

The recoveries of ANT without LMS in the investigated standard samples averaged 99.8–105.6% at post procedure.

### Quantification of ANT and ANQ using HPLC

ANT and ANQ concentration were quantified using HPLC (LC-16, Shimadzu) equipped with WondaSil C_18_-WR (length × I.D.: 15 cm × 4.6 mm × 5 μm particles, GL sciences inc.) and UV–visible detector (SPD-16, Shimadzu). Isocratic elution was performed as follows: acetonitrile and water (62.5: 37.5, v: v) at a flow rate of 1.0 mL min^−1^. Chromatography was performed at 40 °C, ANT and ANQ were detected at 254 nm, using 5 μL injection volume.

### Instrumental analysis by GC/MS

To determine the possible transformation products of ANT with and without Tween 80 or Tween 20 in LMS-HOBt, the reaction solution analyzed by GC/MS containing 20 mg L^−1^ ANT, 600 mg L^−1^ Tween 80 or Tween 20, 2 mM HOBt, and 1.88 U mL^−1^ laccase in 0.2 M sodium acetate, at pH 4.5. Control samples with and without Tween 80 or Tween 20 were also analyzed by GC/MS for comparison. After 24 h incubation, the reaction solution was subjected to solid-phase extraction (SPE) on C_18_ column (6 mL, 500 mg) that had been preconditioned by sequential rinses with 10 mL *n*-hexane, followed by methanol and water at a flow rate of 10 mL min^−1^. After sample loading, the column was rinsed with 10 mL water and drained for 10 min before being eluted with 10 mL *n*-hexane. The elution was collected and constituted to 1.5 mL for GC/MS analysis.

## Results and discussion

### Effect of Tween 80 on ANT transformation in LMS-HOBt

Preliminary results have shown that: (i) Due to the low oxidation–reduction potential of fungal laccase, the conversion effect of ANT is not obvious, but it still has a kind of promoting effect; the production of ANQ, the conversion product of ANT, has been observed throughout the degradation process, which can also explain a single lacquer Enzymes can convert ANT. Laccase weak ability in ANT transformation, which is minimally enhanced by Tween 80 without HOBt (Figure.S1); (ii) Tween 80 within 2.5 CMC has little effect on increasing the conversion rate of ANT, indicating that low concentration of Tween 80 does not have a good promotion effect, while at a concentration above 2.5 CMC, the degradation rate enhances with the concentration of Tween 80 increasing gradually and the production rate of ANQ also gradually increases. However, without the addition of Tween 80, enhancement of ANT transformation in LMS-HOBt is observed (Figure. S1 and S2). This confirms the crucial role HOBt plays in ANT transformation.

In fact, the solubility of ANT in solution is extremely low. Within 1 CMC Tween 80, the solubility of ANT is 0.024 mg L^−1^. As the concentration of Tween 80 increases, the solubility of ANT also increases. When the concentration of Tween 80 is 40 CMC, the solubility of ANT is only 0.319 mg L^−1^. In addition, ANT is a kind of highly hydrophobic compound. In the process of ANT degradation, the generated ANQ will compete with ANT for the position of the micelle space, resulting in less ANT dissolution. So the amount of ANT dissolved will be less in the actual enzymatic reaction liquid.

ANT is dissolved in the buffer solution containing with Tween 80 for 24 h to reach the solubilization equilibrium, and then LMS-HOBt is added to degrade ANT for 24 h (Figure. S2). From Figure. S3, it can be seen that the solubility of ANT does increase after 24 h, but it is less than 0.5 mg L^−1^ in the end. When the concentration of Tween 80 is 40 CMC, the solubility of ANT is only 0.319 mg L^−1^ , while LMS-HOBt converts 20 mg L^−1^ ANT to ANQ. So solubility and degradation rate are not necessarily related. The former is the physical properties of the compound, while the latter reflects the chemical reaction. Therefore, the increase in solubility of ANT caused by Tween 80 is not the main reason for promoting the conversion of ANT.

**Figure 1:**
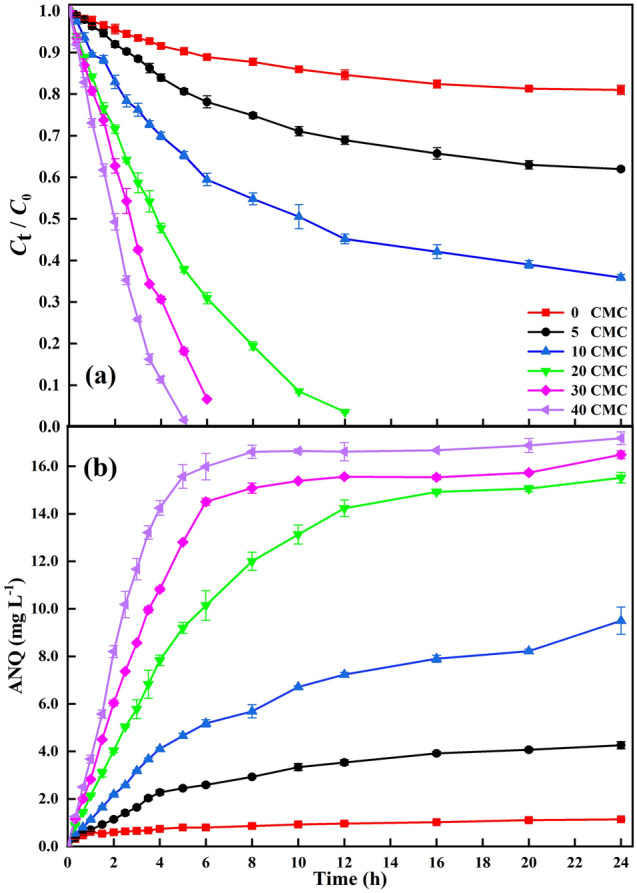
The degradation of ANT and the production of ANQ within 24 h changed with time. Transformation of 20 mg L^-1^ ANT and accumulation of ANQ by 1.88 U mL^-1^ laccase with 2 mM HOBt in the presence of different concentrations of Tween 80 in 0.2 M sodium acetate solutions, pH 4.5 at 30 °C after 24 h incubation period: (**a**) Transformation rates of ANT by LMS-HOBt in the presence of Tween 80; (**b**) Accumulation of ANQ according to (**a**). Error bar represents standard deviations (n = 3).

**Figure 2:**
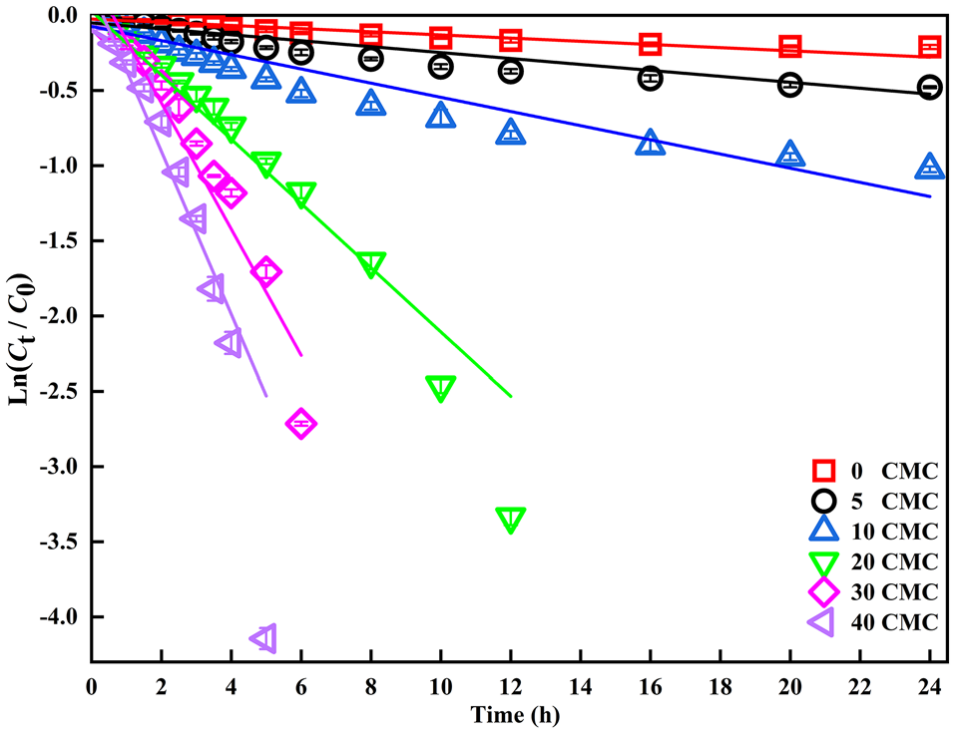
Pseudo-first-order kinetic equation for the conversion of ANT by LMS-HOBt.

To investigate the effect of Tween 80 on ANT transformation, different concentrations of Tween 80 were added to ANT and LMS-HOBt solution. As shown in Fig. 1a, the enhancement of ANT transformation during 24 h incubation period depended on Tween 80 concentration, accordingly, concentration of ANQ is shown in Fig. 1b. The results show that 95% conversion of ANT is obtained with Tween 80 above 20 CMC in LMS-HOBt, producing the corresponding ANQ product in > 13 mg within 12 h. At Tween 80 of 40 CMC, the conversion rate of ANT reaches almost 100%, and generates 17.18 mg L^−1^ of ANQ. Furthermore, the transformation of ANT over the entire procedure was fitted to the apparent pseudo first-order rate equation (Fig. 2), and the transformation rate constants (*k*) and half-lives (*T*_1/2_) calculated are shown in Table 1. Notably, *k* value of ANT transformation is approx 50.05 times higher in the presence of Tween 80 (40 CMC) than in its absence, with half-lives of 1.30 h and 65.86 h, respectively (Table 1). This highlights that the presence of Tween 80 enhances ANT transformation rate. Correspondingly, the accumulation rate of ANQ increases with increasing Tween 80 concentration (Fig. 1b). Additionally, the rate turning point of ANT transformation and ANQ accumulation occurs at 10 CMC Tween 80. Hence, Tween 80 is a major factor influencing LMS-HOBt transformation process of ANT in aqueous solution. Yulong et al.^52^ reported that the degradation rate of anthracene by Trametes versicolor laccase after 72 h of culture was 7%, and the conversion rate of anthracene by the modified laccase increased to 36%. The Coriolopsis gallica UAMH 8260 purified laccase reported by Michael et al.^53^ converted 22% of 20 μM anthracene within 6 h. Finally, the conversion rate of 1 μg·L^−1^ anthracene after 24 h degradation by laccase, also purchased from Sigma-Aldrich, is less than 30%^54^. Based on the above reports, it can be found that the addition of redox media is necessary. Under the Tween 80 in LMS-HOBt, the degradation of ANT is significantly improved.

**Table 1:** The pseudo-first order kinetic parameters of ANT conversion by LMS-HOBt.

Concentrations	Measured parameters		
of Tween 80	*k* (h^−1^)	*T*_1/2_ (h)	*R*^2^
0 CMC	0.0106 ± 0.0009	65.8661 ± 5.5924	> 0.9019
5 CMC	0.0198 ± 0.0015	35.2095 ± 2.6674	> 0.9114
10 CMC	0.0472 ± 0.0038	14.7811 ± 1.1900	> 0.9041
20 CMC	0.2135 ± 0.0117	3.2564 ± 0.1785	> 0.9622
30 CMC	0.4129 ± 0.0381	1.6931 ± 0.1562	> 0.9231
40 CMC	0.5411 ± 0.0604	1.2972 ± 0.1448	> 0.8981

Experimental apparent pseudo-first-order kinetic parameters (*k*) and half-lives (*T*_1/2_) values determined for LMS-HOBt transformation of ANT with different Tween 80 concentrations corresponding to Fig. 1a.

As previously mentioned, the lipid peroxidation reactions caused by HAT can enhance PAHs transformation, as determined by the detection of TBARS (thiobarbituric acid-reactive substances) from unsaturated fatty acids^8,9,34^. For example, inhibition of fluorine oxidation by free-radical scavenger butylated hydroxytoluene, shows that the oxidation process is a consequence of lipid peroxidation mediated by *P. chrysosporium* manganese peroxidase^35^. Furthermore, similar enhancements can also occur in the oxidation of other large molecular substrates, such as biodegradation of lignin by linoleic acid, arachidonic acid, and Tween 80 or Tween 20^36–39^, cellulose digestibility^40,41^ and biobleaching hardwood kraft pulp^42^. Additionally, degradation of other organic pollutants can also be enhanced by Triton X-100, including indole^43^, bisphenol A^44,45^, phenol^46–48^, fungicide miconazole and antidepressant^49^, and asphalt^50^. As a result of lipid peroxidation reactions, the unstable *α*-position of ether oxygen bond (–C–O–C–) in Tween 20, carbon–carbon double bond (C=C) and that in Tween 80 should be also considered. The structure of Tween 80 and 20 are presented in Figure. S10.

**Figure 3:**
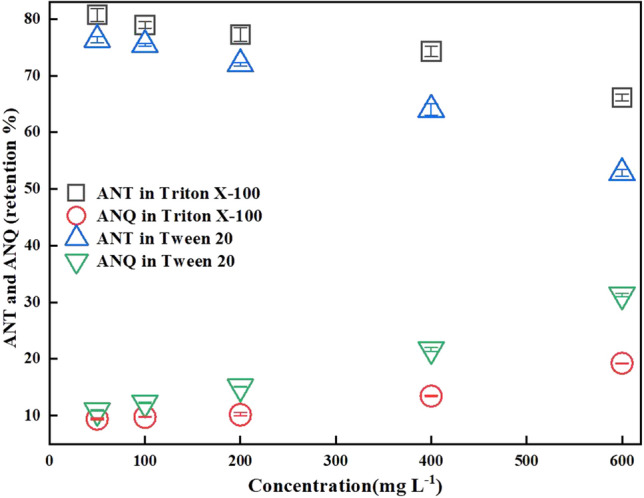
Transformation of 20 mg L^-1^ ANT and accumulation of ANQ by 1.88 U mL^-1^ laccase with 2 mM HOBt in the presence of different concentrations of Tween 20 and Triton X-100 in 0.2 M sodium acetate solutions, pH 4.5 at 30 °C after 24 h incubation period. Error bar represents standard deviations (n = 3).

As illustrated in Fig. 3, the generation of main product ANQ by ANT degradation in LMS-HOBt increases with increasing Tween 20 and Triton X-100 concentration after 24 h. In this case Tween 20, it displayed greater enhancement compared with Triton X-100.

### Identification of ANT transformation products

Transformation products of ANT, with and without Tween 80 (Table 2) or Tween 20 (Table 3) in LMS-HOBt, were identified using GC/MS.

**Table 2:** Information of peak at the same time by possible products in both systems.

No	Peak areas observed in aqueous solution			Possible structure	Formula	Degree of match/%
	Tween 80	LMS-HOBt				
		With Tween 80	Without Tween 80			
1	1.75E + 05	2.25E + 06	–		C_8_H_16_O	52.3
2	–	3.63E + 05	–		C_8_H_14_O	53.5
3	–	9.09E + 05	–		C_7_H_14_O_2_	65.1
4	1.01E + 05	1.27E + 06	–		C_9_H_18_O	52.7
5	–	4.60E + 05	–		C_9_H_18_O_2_	64.7
6	–	1.96E + 05	–		C_9_H_16_O	53.8
7	2.83E + 05	1.37E + 07	–		C_8_H_16_O_2_	63.3
8	–	5.71E + 05	–		C_10_H_20_O_2_	59.6
9	1.53E + 05	4.68E + 05	–		C_10_H_18_O	70.1
10	1.11E + 05	1.44E + 07	–		C_9_H_18_O_2_	61.9
11	–	1.25E + 06	–		C_8_H_14_O	66.4
12	2.99E + 05	3.41E + 05	–		C_11_H_20_O	71.0
13	–	4.28E + 05	–		C_10_H_18_O_2_	73.3
14	–	5.35E + 05	–		C_11_H_20_O_2_	68.5
15	–	5.93E + 05	–		C_15_H_30_O_2_	73.5
16	–	1.01E + 06	8.73E + 07		C_14_H_10_	98.5
17	5.07E + 05	7.42E + 06	–		C_16_H_32_O_2_	83.2
18	–	7.20E + 07	1.06E + 07		C_14_H_8_O_2_	97.5
19	1.03E + 05	2.07E + 06	–		C_18_H_36_O_2_	77.4
20	–	1.29E + 06	–		C_18_H_36_O_3_	71.7

Peak areas observed by GC/MS and possible formula of products produced with or without Tween 80 in LMS-HOBt and Tween 80 alone. Experimental conditions: *C*_ANT_ = 20 mg L^−1^, *C*_Tween 80_ = 40 mg L^−1^, *C*_laccase_ = 1.88 U mL^−1^, *C*_HOBt_ = 2 mM.

**Table 3:** Information of peak at the same time by possible products in both systems.

No	Peak areas observed in aqueous solution			Possible structure	Formula	Degree of match/%
	Tween 20	LMS-HOBt				
		With Tween 20	Without Tween 20			
1	–	2.85E + 06	–		C_11_H_22_O_2_	68.4
2	–	2.85E + 06	–		C_10_H_20_O_2_	59.6
3	–	2.80E + 07	–		C_13_H_26_O_2_	77.4
4	–	2.49E + 07	–		C_12_H_24_O_2_	67.1
5	–	1.33E + 07	–		C_15_H_30_O_2_	73.5
6	–	6.25E + 06	–		C_14_H_28_O_2_	85.7
7	–	5.19E + 07	8.73E + 07		C_14_H_10_	98.3
8	–	5.38E + 05	–		C_19_H_34_O_6_	51.9
9	–	6.21E + 06	–		C_17_H_34_O_2_	56.7
10	–	6.11E + 06	–		C_16_H_32_O_2_	83.2
11	–	2.66E + 07	1.06E + 07		C_14_H_8_O_2_	97.2
12	–	2.96E + 05	–		C_16_H_32_O_3_	71.9
13	–	5.64E + 05	–		C_16_H_32_O_4_	85.3
14	–	8.19E + 05	–		C_18_H_36_O_2_	77.4

Peak areas observed by GC/MS and possible formula of products produced with or without Tween 20 in LMS-HOBt and Tween 20 alone. Experimental conditions: *C*_ANT_ = 20 mg L^−1^, *C*_Tween 80_ = 40 mg L^−1^, *C*_laccase_ = 1.88 U mL^−1^, *C*_HOBt_ = 2 mM.

### Degradation pathway of ANT in LMS-HOBt with Tween 80

As shown in Figure. S5 and Figure. S6, the degradation rate of ANT and ANQ production without Tween 80-LMS-HOBt are less than that of Tween 80-LMS-HOBt with 600 mg L^−1^, hence, Tween 80 significantly influences the conversion of ANT in LMS-HOBt. Additionally, in order to investigate the effect of Tween 80 on the conversion of ANT to other products under the same conditions, the products from two systems and blank control of Tween 80 were compared (Table 2). Auto-oxidation occurs in the blank control, consisting of aldehyde and acid products. Compared with Tween 80-LMS-HOBt, a large number of these acid, aldehyde, and ester products are observed. Furthermore, the corresponding chromatographic peak areas increase twofold by GC/MS analysis, indicating that LMS-HOBt can promote the oxidation of Tween 80. In addition, the structural formula of Tween 80 contains a large number of ether bonds, which are not present in the products, and indicates that they have been oxidized.

### Degradation pathway of ANT in LMS-HOBt with Tween 20

In order to verify the oxidation of the ether bonds, Tween 20, which is bereft of C=C bonds and possesses only ether-oxygen bonds, was investigated for the degradation of ANT products in LMS-HOBt.

The enzymatic reaction solution after 24 h of degradation with and without Tween 20 were analyzed by GC/MS under the same experimental conditions as Tween 80. The obtained analytical data was examined for possible products by NIST MS Search (Version 2.2). The chromatogram of LMS-HOBt without Tween 20 is shown in Figure. S5.

As shown in Figure. S5 and Figure. S8, the degradation rate of ANT and ANQ production in LMS-HOBt without Tween 20 are less than that in Tween 80-LMS-HOBt with 600 mg L^−1^, indicating that Tween 20 has little influence on the main product, hence, the main conversion products are ANQs. The effect of Tween 20 on the conversion of LMS-HOBt from ANT to other products was examined by comparing with corresponding systems. Table 3 shows that the retention time of ANT and ANQ conversion products are 15.20 min and 17.07 min, respectively. However, numerous acid, olefin, and ester products containing between 8–17 carbons are observed in both Tween 20-LMS-HOBt and blank control Tween 20, which are not present in LMS-HOBt. It was speculated that Tween 20 underwent degradation during the conversion of Tween 20-LMS-HOBt to ANT, but no auto-oxidation evidence in Tween 20 was found. It is well known that Tween 20 contains ether oxygen bonds, except C=C bonds. Tween 80 contains both ether oxygen bonds and carbon–carbon double bonds. Hence, Tween 20 is more stable than Tween 80. However, such structural differences could not produce aldehydes in Tween 20-LMS-HOBt system, and the absence of ether bonds in the product suggest that those in Tween 20 were oxidized.

### Effect of Tween 80 on ANT degradation enzyme activity in LMS-HOBt

**Figure 4:**
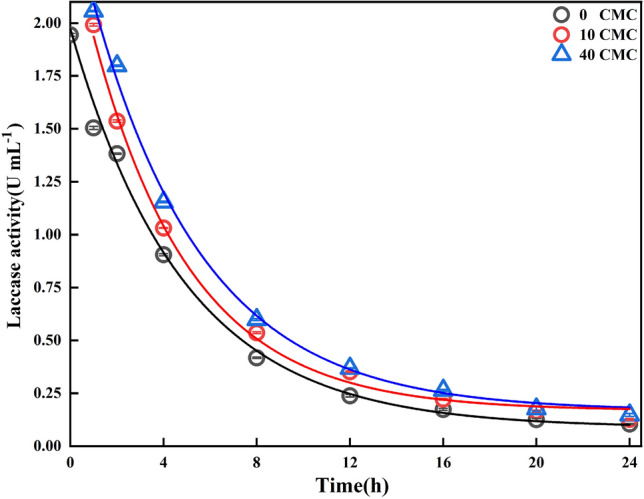
Changes of laccase activity in LMS-HOBt at different times.

In order to investigate the effect of Tween 80 addition on laccase activity during ANT degradation in LMS-HOBt, laccase activity was measured at different times (0–24 h at 4 h intervals) during the conversion process (Fig. 4).

As the enzymatic reaction progressed, laccase activity decreases. However, the addition of Tween 80 promotes an increase in activity compared to that without Tween 80. Moreover, as the concentration of Tween 80 increases from 10 to 40 CMC, laccase activity shows a significant increase, indicating that the addition of Tween 80 increases laccase activity in LMS-HOBt, thereby increasing ANT conversion rate. Prolongation of the enzymatic reaction to 1 h, generates 1.5038 U·mL^−1^ laccase activity in the system without the addition of Tween 80 and 1.9916 U·mL^−1^ with 10 CMC Tween 80, as well as a gradual increase being observed for 40 CMC Tween 80 with a laccase activity of 2.0563 U·mL^−1^. Therefore, during the entire enzymatic reaction, the laccase activity in the system could be continuously enhanced by increasing Tween 80 concentration.

### Mechanism analysis of ANT degradation in Tween 80-LMS-HOBt

According to the results presented in Sect. 3.2.1 and 3.2.2, the degradation products of Tween 80 in LMS-HOBt have been determined, with Tween 80 and ANT being co-converted during ANT conversion in Tween 80-LMS-HOBt. Specifically, the active groups produced by Tween 80 promote the conversion of ANT.

Since the main hydrophobic structure of Tween 80 consists of unsaturated aliphatic hydrocarbons containing carbon–carbon double bonds, the hydrogen atoms are more active and easily oxidized. Reports have shown that enhanced conversion of PAHs and lignin is obtained with lipid peroxidation^51^, and that the unsaturated aliphatic hydrocarbons could co-convert with the target degradation products.

**Figure 5:**
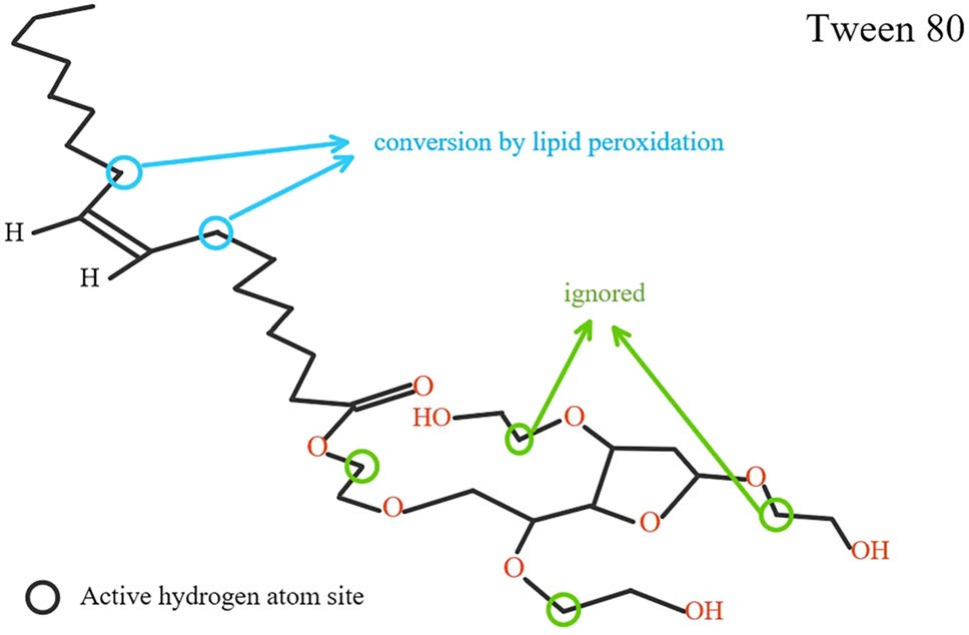
Active hydrogen locus in the tween 80.

In summary, analysis of the degradation products by GC/MS and the presence of C=C bonds in the degradation products did not reveal the presence of ether-oxygen bonds. However, Tween 80 has only one C=C bond but is rich in ether-oxygen bonds. Therefore, Tween 80 can possibly enhance the conversion of LMS-HOBt to ANT by not only lipid peroxidation, but also the formation of a large number of peroxidation and hydroperoxy radicals, generated by cleavage of ether-oxygen bonds, which enhances the ANT degradation. However, although Tween 80 can enhance ANT conversion to ANQ, it can also hinder further conversion of ANQ. In addition, reported studies^28^ have confirmed that both Tween 80 and Tween 20 can occur auto-oxidation reaction, even though Tween 20 is bereft of C=C bonds. Therefore, this study highlights that existing reports have ignored the conversion of ANT due to peroxidation of α–C–H on the ether oxygen bond, as shown in Fig. 5.

**Figure 6:**
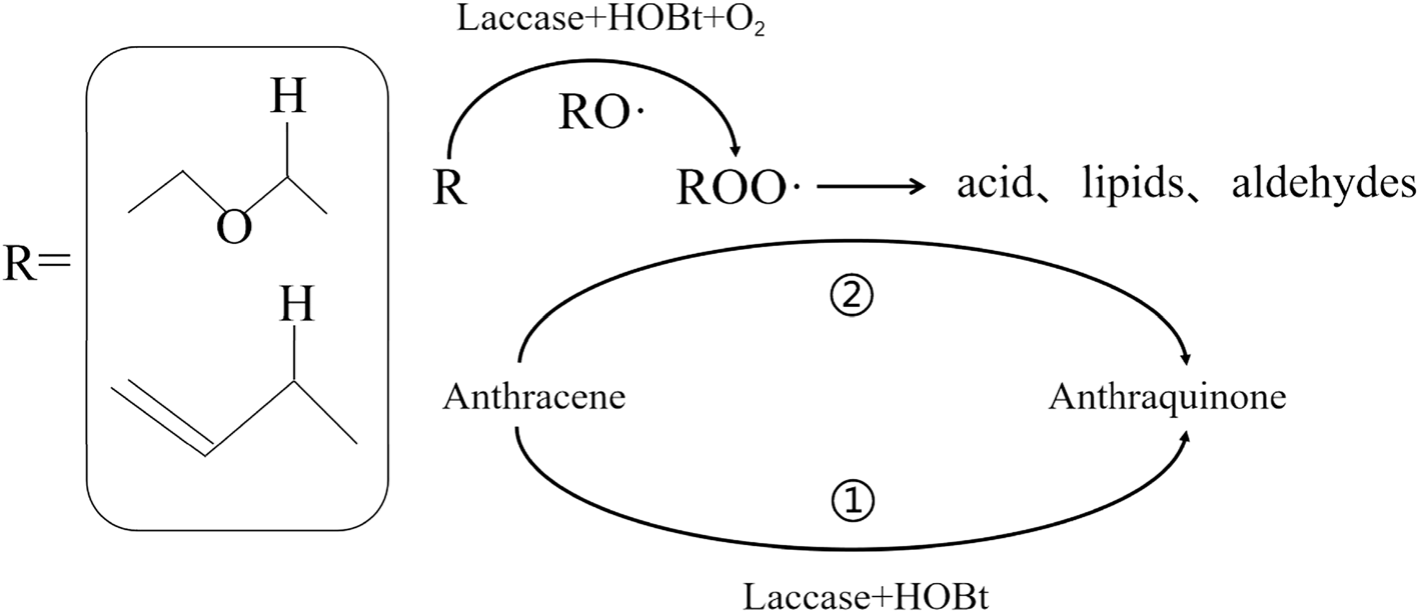
Mechanism of degradation of ANT enhanced by Tween 80-LMS-HOBt.

Combined with the reported^28^ auto-oxidation processes of Tween 80 and Tween 20, the transformation path of ANT enhanced by Tween 80 and mediated by laccase medium (HAT mechanism) is speculated roughly as follows: The hydrogen (α–C–H of ether-oxygen bond and C=C bond) of Tween 80 is removed by the laccase-medium system, promoting degradation of Tween 80 and generation of numerous reactive oxygen species (ROS) such as RO• and ROO•, which oxidize Tween 80 producing free radicals, while converting ANT. This free radical chain reaction proceeds to the end (Fig. 6).

## Conclusions

In this study, laccase systems mediated by HAT mechanism in HOBt media enhance ANT conversion. ANT degradation with two different laccase-medium systems with either Tween 80 or Tween 20, as well as the blank control was analyzed. The results show that ANT degradation via laccase-medium system (LMS-HOBt) with the addition of Tween 80 complies with the pseudo first order kinetic equation. The kinetic parameter -*k* (h^−1^) and corresponding half-life *T*_*1/2*_ decrease with increasing Tween 80 concentration between 0–40 CMC. Additionally, LMS-HOBt has weak promotion effect on the degradation of ANT. Tween 80 and ANT have been co-converted during ANT conversion in Tween 80-LMS-HOBt. In the LMS-HOBt system with Tween 80/Tween 20, the degradation of Tween 80/Tween 20 generate a large number of ROS, which continue to oxidize Tween 80/Tween 20 while converting ANT. Although both ROS and LMS-HOBt can convert ANT to ANQ, ROS will be more efficient than LMS-HOBt. This mechanism of enhanced transformation may provide new methods for the degradation of PAHs.

## Supplementary Information

Supplementary Information.
