# Supplementary material for: Enhancement and analysis of Anthracene degradation by Tween 80 in LMS-HOBt
Source: Sci Rep. 2021 Jun 23;11:13121. doi: 10.1038/s41598-021-90609-5 (PMC8222252; doi:10.1038/s41598-021-90609-5)
Supplement: Supplementary file 1 — Supplementary Information. [file 41598_2021_90609_MOESM1_ESM.doc]

**Supporting Information**

**Enhancement and analysis of Anthracene degradation by Tween 80 in LMS-HOBt**

Zuoyi Yang**^a^** , Xingchen Mao**^a^**^*^ , Jiahao Cui**^a^** , Yujie Wang**^a^** , Yaping Zhang**^a^**

**^a^**Guangzhou Key Laboratory of Environmental Catalysis and Pollution Control, School of Environment Science and Engineering, Guangdong University of Technology, Guangzhou 510006, China

***Corresponding author and contact details**

**Xingchen Mao**,

Mailing address:Guangzhou Key Laboratory of Environmental Catalysis and Pollution Control, School of Environment Science and Engineering, Guangdong University of Technology, Guangzhou 510006, China

Tel: +86-18871850109

E-mail: mao-xingchen@outlook.com

Submitted to

**Scientific Reports**

Pages: 14

Figures: 12

1. **Effect of Tween 80 on ANT transformation by laccase**

**Fig. S1.** Transformation of 20 mg L^-1^ ANT by 1.88 U mL^-1^ laccase alone and a series of multiple CMC Tween 80 in pH 4.5, 0.2 M sodium acetate solutions at 30℃ after an incubation period of 24 h. A remaining ANT and ANQ as a percentage of the inatial concentration (*C*_0_), Let *C*_0_ (ANQ) be 20 mg L^-1^. Error bar represents standard deviations (n = 3).

.

1. **Effect of Tween 80 on ANT transformation by LMS-HOBt**
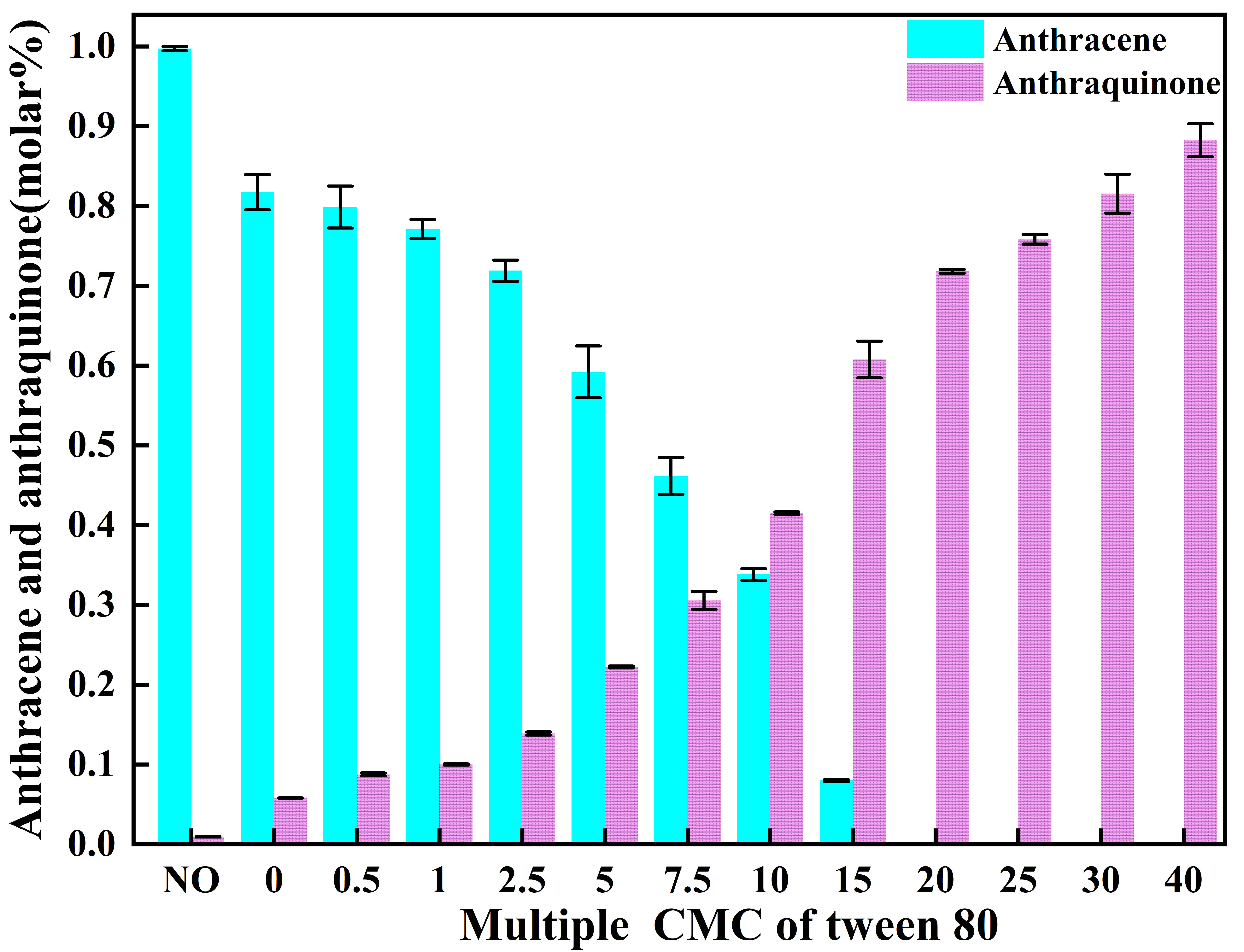


**Fig. S2.** Transformation of 20 mg L^-1^ ANT by 1.88 U mL^-1^ laccase and 2 mM HOBT in the presence of a series of multiple CMC Tween 80 in 0.2 M, pH 4.5 sodium acetate solutions at 30℃ after an incubation period of 24 h. A remaining ANT and ANQ as a percentage of the inatial concentration (*C*_0_), Let *C*_0_ (ANQ) be 20 mg L^-1^. Error bar represents standard deviations (n = 3).


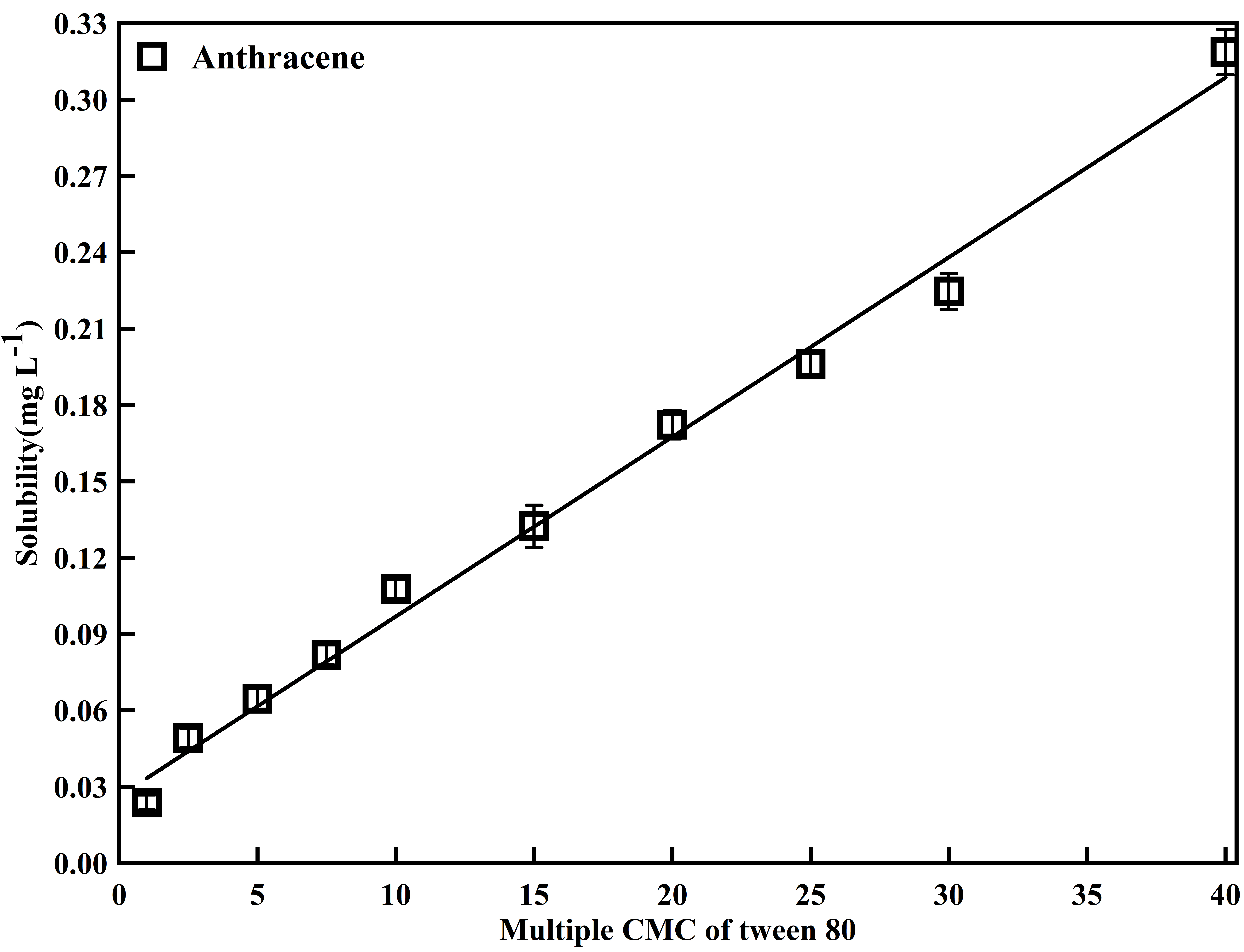


**Fig. S3.** The solubility of ANT in Tween 80

1. **Apparent pseudo first-order rate equation**


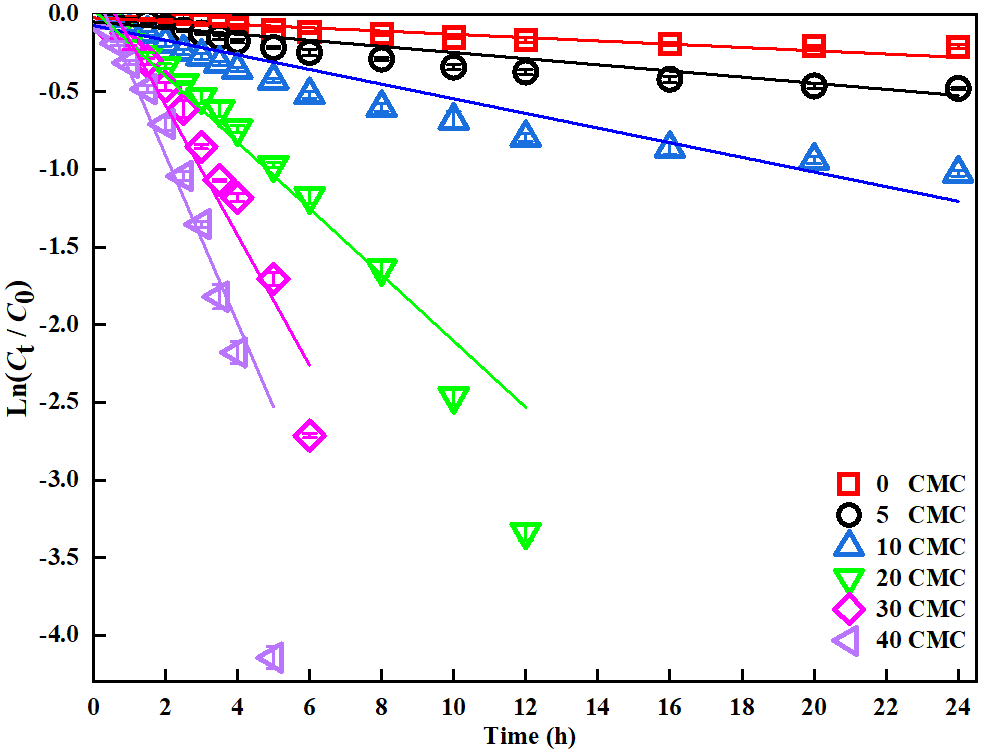


**Fig. S4.** Fitting of the ANT transformation data during the entire procedure in Fig. 1a to the apparent pseudo-first-order rate equation. Error bar represents standard deviations (n = 3).

1. **The degradation pathway of ANT in LMS-HOBt by adding Tween 80**


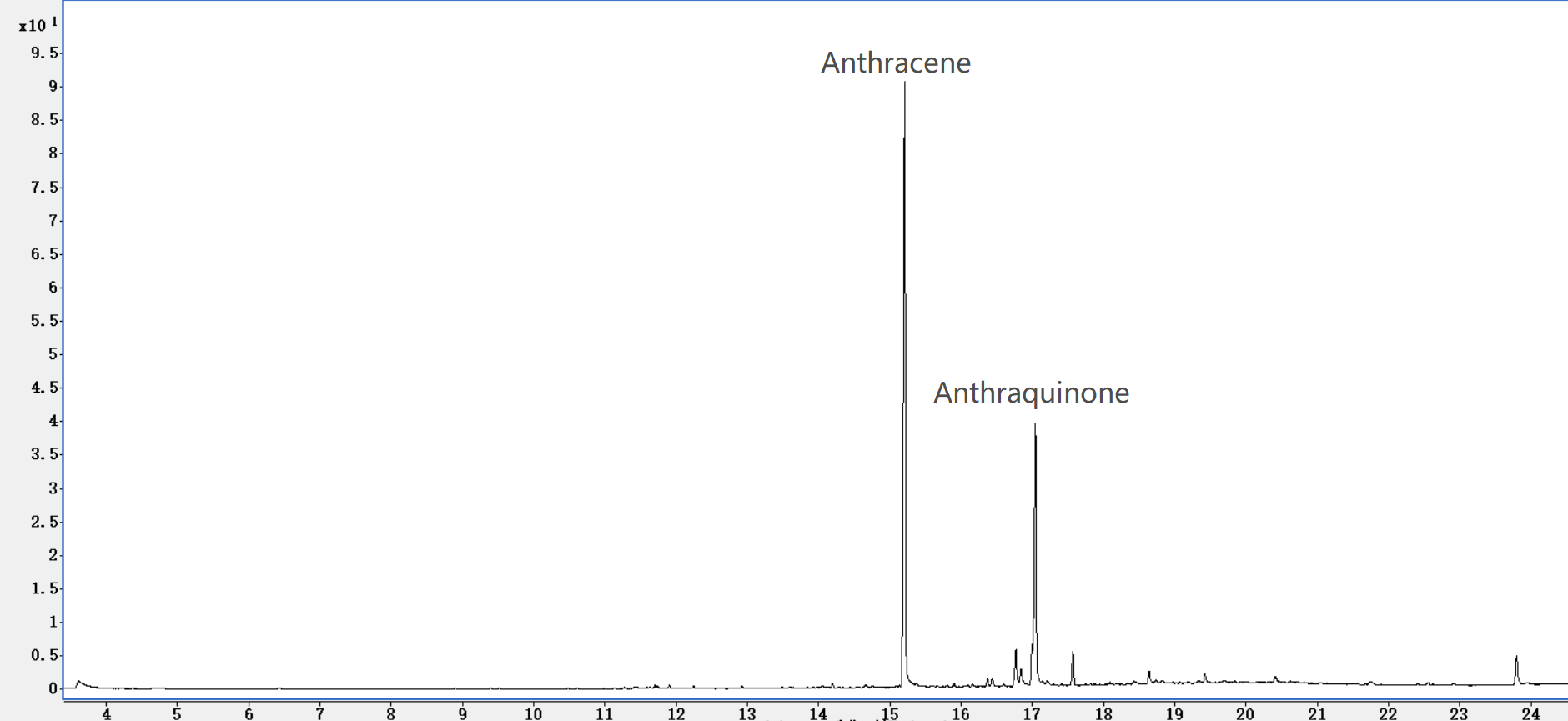


**Fig. S5.** Chromatogram of ANT transformed by LMS-HOBt for 24 h


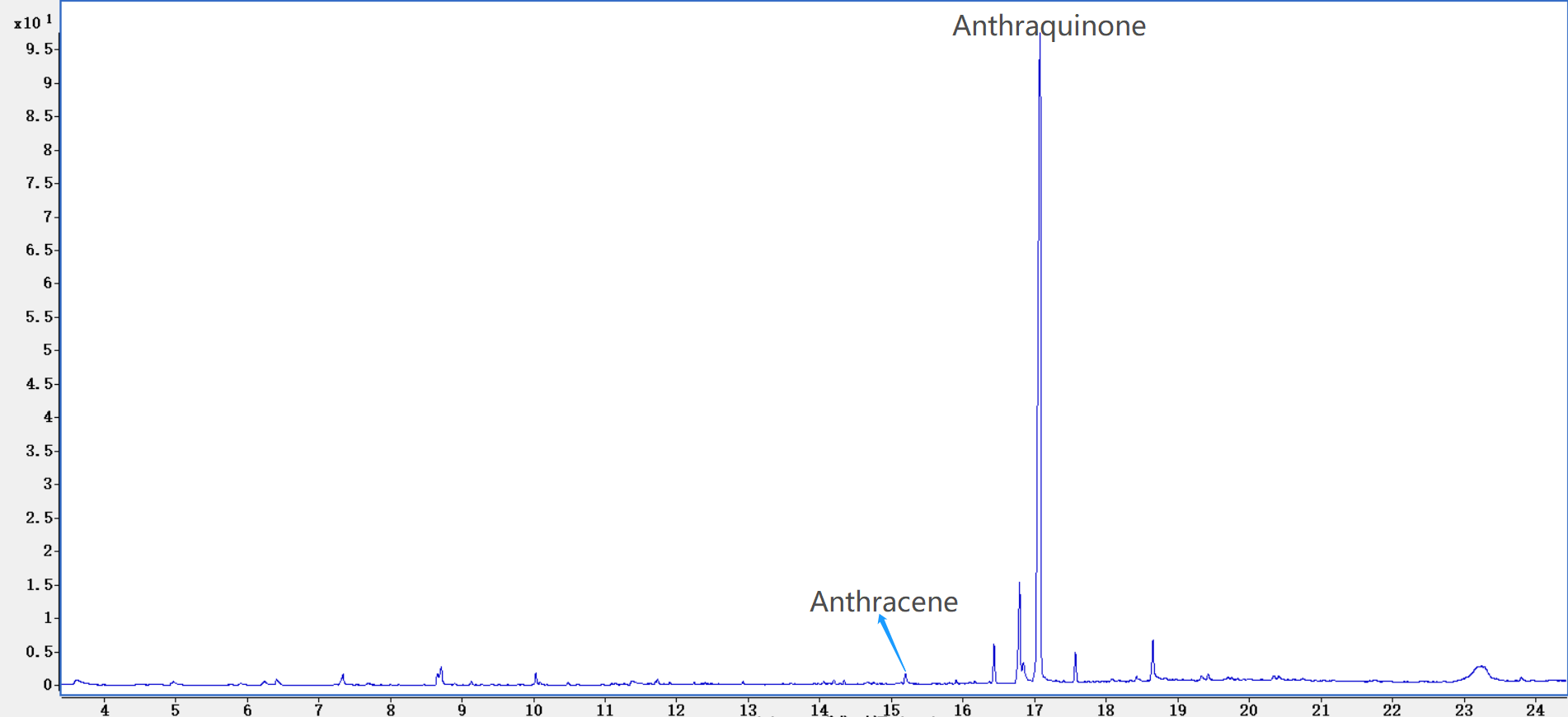


**Fig. S6.** Chromatogram of ANT transformed by Tweeen 80-LMS-HOBt for 24 h


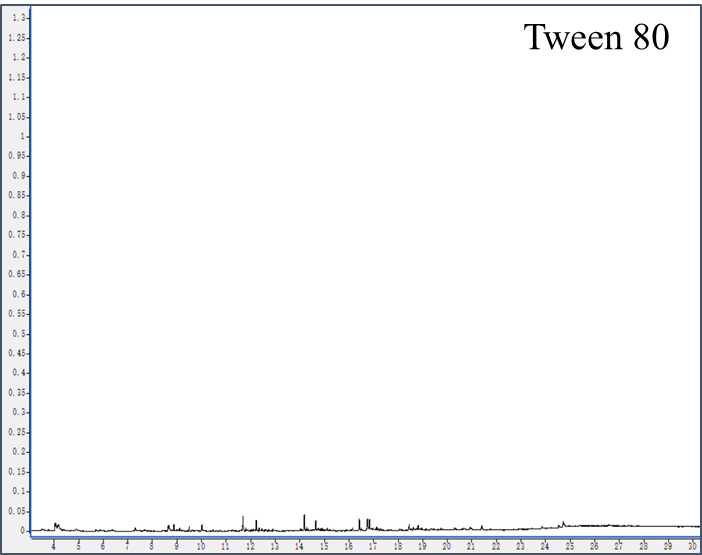


**Fig. S7.** Chromatography of Tween 80 in the same experimental condition

1. **The degradation pathway of ANT in LMS-HOBt by adding Tween 20**


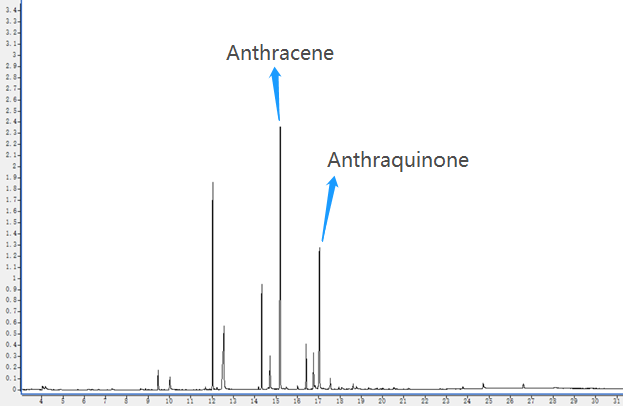


**Fig. S8.** Chromatogram of ANT transformed by Tween 20-LMS-HOBt for 24 h


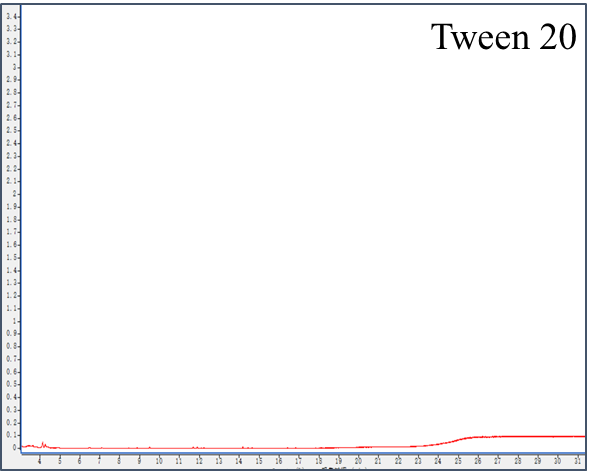


**Fig. S9.** Chromatography of Tween 20 in the same experimental condition

1. **Structural formula of Tween 80 and Tween 20**


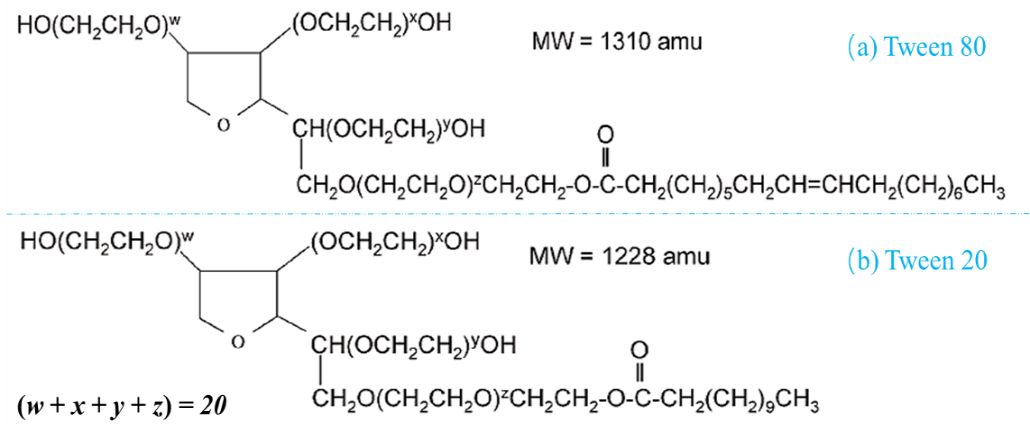
**Fig. S10.** Structural formula of Tween 80 and Tween 20 ^1^: (a) Tween 80, (b) Tween 20. Total number (w + x + y + z) equals 20.

1. **Transformation products of ANT retrieved by NIST MS Search 2.2**

**Fig. S11 (No.1-20).** Possible structure of ANT transformation products with or without Tween 80 in LMS-HOBt and Tween 80 alone


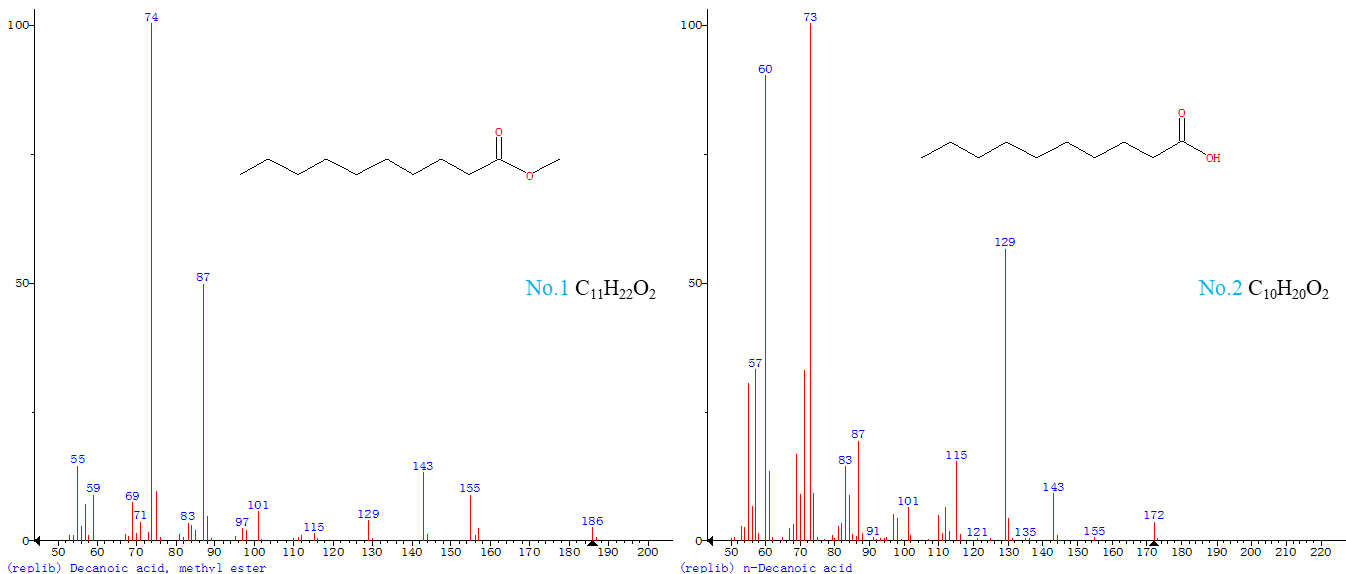


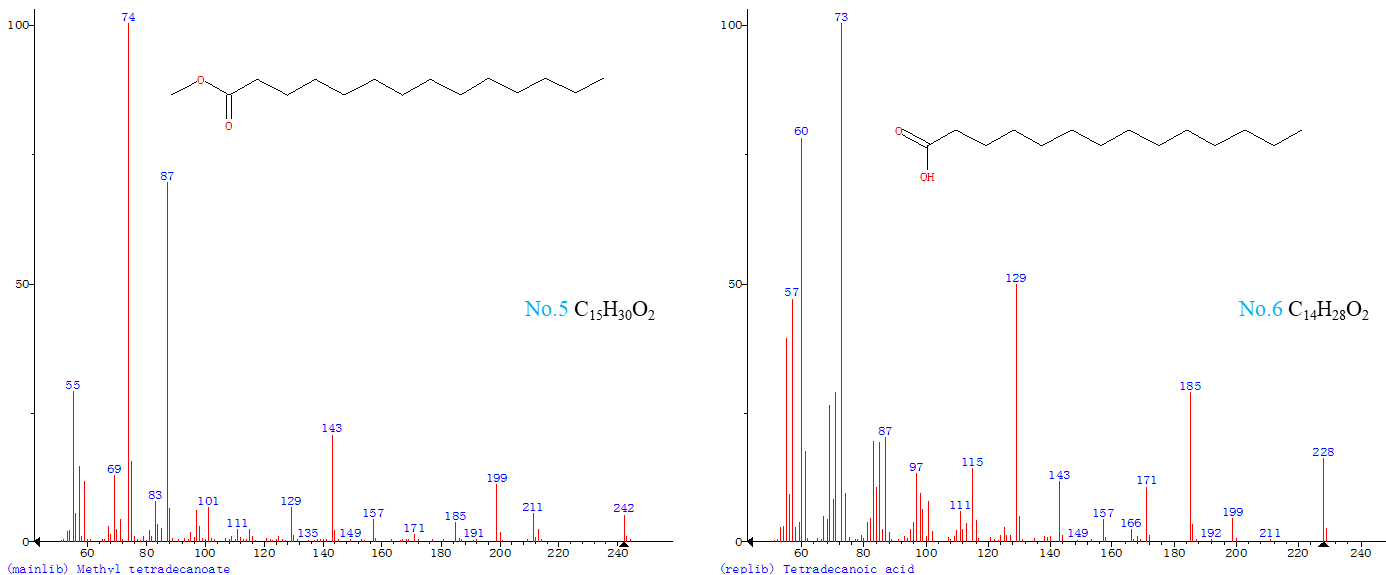


**Fig. S12 (No.1-14).** Possible structure of ANT transformation products with or without Tween 20 in LMS-HOBt and Tween 20 alone

**References**

1. Kerwin, B. A. Polysorbates 20 and 80 used in the formulation of protein biotherapeutics: Structure and degradation pathways. *Journal of Pharmaceutical Sciences* **97**, 2924-2935, DOI: <https://doi.org/10.1002/jps.21190> (2008).
